# Chondroprotective effects of platelet lysate towards monoiodoacetate-induced arthritis by suppression of TNF-α-induced activation of NF-ĸB pathway in chondrocytes

**DOI:** 10.18632/aging.101952

**Published:** 2019-05-14

**Authors:** Li Yan, Li Zhou, Danting Xie, Wenxi Du, Fangming Chen, Qiang Yuan, Peijian Tong, Letian Shan, Thomas Efferth

**Affiliations:** 1 The First Affiliated Hospital, Zhejiang Chinese Medical University, Hangzhou, China; 2 Center for Stem Cell Translational Research, Zhejiang Chinese Medical University, Hangzhou, China; 3 Academy of Chinese Medical Sciences, Zhejiang Chinese Medical University, Hangzhou, China; 4 College of Pharmacy, Zhejiang Chinese Medical University, Hangzhou, China; 5 Department of Pharmaceutical Biology, Institute of Pharmacy and Biochemistry, Johannes Gutenberg University, Mainz, Germany; * Equal contribution

**Keywords:** platelet lysate, growth factor, osteoarthritis, TNF-α, NF-κB

## Abstract

Platelet lysate (PL) contains a cocktail of growth factors that actively participates in cartilage repair. This study was designed to determine the effect and mechanism of PL on osteoarthritis (OA). An arthritis model was established to mimic human OA by intra-articular injection of monoiodoacetate (MIA) to Sprague Dawley (SD) rats. The model was weekly treated with PL by intra-articular injection. Thermal withdrawal latency, mechanical withdrawal threshold, and treadmill gait were tested for pain behavior observation. Histopathological and immunohistochemical analyses were conducted for evaluating cartilage degradation. Real time PCRs and Western blots were conducted to elucidate the mechanism of PL on primary chondrocytes. Results showed that, *in vivo*, PL significantly attenuated pain symptoms and exerted chondrocyte-protective and extracellular matrix (ECM)-modifying effect on the arthritic cartilage in a dose-dependent manner. The *in situ* expressions of type II Collagen (Col2) and matrix metalloproteinase 13 (Mmp13) in the arthritic cartilage was abnormal and was restored by PL. *In vitro*, PL significantly restored tumor necrosis factor α (TNF-α)-suppressed anabolic gene expression (*Col2* and *aggrecan*) and TNF-α-increased catabolic gene expression (*Col10*, *Mmp13*, *Adamts5*, and *Adamts9*) in chondrocytes. The effects were mediated by TNF-α downstream signaling, including inhibition of NF-κB and c-Jun activities. This study provides certain knowledge of anti-OA effect and TNF signaling-related mechanism of PL, placing it as a promising and alternative option for OA therapy in the future.

## INTRODUCTION

Osteoarthritis (OA) of the knee is the most common joint disease characterized by progressive destruction of articular cartilage, thinning and eventual wearing of cartilage, thus resulting in joint pain and limited joint movement [1]. The pain and joint dysfunction of OA place a major burden on communities as well as health and social care systems, making OA a leading cause of global disability [2]. It is estimated that 15% of the world populations suffer from OA, including more than 39 million Europeans and more than 20 million Americans [3]. By 2020, these numbers will probably be doubled [4]. Due to the limitation of intrinsic self-repairing capacity of the load-bearing articular cartilage of joint, even minor lesions or injuries may lead to progressive damage, joint degeneration, and finally OA [1]. Many therapeutic efforts have been made for OA treatment. Surgical approaches, such as knee arthroplasty, are successfully used for addressing end-stage OA. However, adverse outcomes have some possibility to occur with the surgeries, including high cost and limited lifespan of prostheses. Preoperative non-surgical approaches, such as intra-articular pharmacological injections, are more important for the treatment of early- and middle-stage OA. This includes both intra-articular corticosteroid and visco-supplementation injections, which seemingly have successful, albeit short-term benefits to OA patients, including pain relief and knee function improvement [5, 6]. However, the clinical practice guidelines of the American Academy of Orthopaedic Surgeons demonstrated inconclusive evidence to recommend for or against corticosteroid and strong evidence against hyaluronic acid (HA) visco-supplementation injections for OA patients [7]. This has led to the emergence of other injectable options for symptom relief and functional improvement in these patients [8].

In the 1980s, human platelet lysate (PL) prepared by repeated freeze/thaw cycles from peripheral blood or platelet concentrates were firstly described to support proliferation of primary and established cell lines and to promote tissue regeneration and wound healing [9, 10]. PLs have a strong growth promoting action on primary articular chondrocytes, which promote growth and sulfated glycosaminoglycan synthesis of articular chondrocytes [11]. The bioactivity of PL was derived from a series of potent bioactive mediators primarily in α-granules of platelet [12]. A variety of growth factors account for these effects, such as platelet derived growth factors (PDGF), transforming growth factor-β (TGF-β), insulin-like growth factor (IGF-1), epidermal growth factor (EGF), and vascular endothelial growth factor (VEGF) [13, 14]. Those anabolic growth factors stimulate chondrocyte synthesis of proteoglycans, aggrecan and type II collagen, induce chondrocyte proliferation, trigger chondrogenic differentiation of mesenchymal stromal cells, and decrease the catabolic effects of cytokines such as interleukin-1 (IL-1) and the matrix metalloproteinases (MMP) [15]. For example, PDGF and IGF-1 are potent inhibitors of IL-1β-mediated apoptosis of chondrocytes in OA [16], and PDGF, TGF-β, IGF-1 and EGF are regulators of cartilage growth, which can improve chondrocyte metabolism [17, 18]. Recently, intra-articularly injected autologous PL has been reported as an efficient method for temporarily managing OA of the distal interphalangeal joint in athletic horses [19]. In addition, intra-articular autologous PL significantly improved the non-normalized Knee Osteoarthritis and Disability Outcome Score (KOOS) of patients with early and intermediate knee OA in an open-label prospective study [20]. This is certainly a clue that PLs containing growth factors have a promising potential for treatment of OA.

To confirm the therapeutic effect and mechanism of PL on OA, we established an animal model of arthritis by intra-articular monoiodoacetate (MIA)-injection and a cellular model of arthritic chondrocytes by TNF-α treatment. MIA induces cartilage degeneration through production of pro-inflammatory cytokines, damage of pain-related sensory innervation of dorsal root ganglia, induction of cell death, disruption of chondrocyte metabolism and decrease of proteoglycan content, which causes rapidly progressing OA with less invasive procedures and enables standardization [21, 22]. The MIA model is commonly used as an arthritis model mimicking human OA for pain assessment, owing to the consistent pain-like responses throughout the modeling period [23]. It provides measurable changes on joint movements, tactile allodynia, inflammation, and progressive cartilage degeneration that represent markers for OA evaluation [24]. Previous studies reported that the MIA model is useful for evaluating the pathology of non-traumatic OA, pain mechanisms, and therapeutic effects on cartilage [25, 26]. Therefore, this model has been recommended as preferred model to study chondroprotective and analgesic agents compared to the existing surgical and spontaneously developing models [27]. Articular cartilage is a conjunctive tissue composed of only one cell type, chondrocytes, enclosed in a self-synthetized extracellular matrix (ECM) [28]. These specific cells represent approximately 1% of the total cartilage volume and are responsible for matrix composition and integrity. Thereby, they confer to mechanical support and joint lubrication of cartilage [28, 29]. During OA development, pro-inflammatory cytokines, such as TNF-α, participate in cartilage degradation through activation of chondrocyte catabolism. Elevated concentrations of TNF-α in synovial fluid have also been demonstrated in patients with knee OA disease progression [30]. Therefore, we applied TNF-α-treated chondrocytes as OA-like *in vitro* model and performed cellular and molecular experiments to evaluate the therapeutic mechanism of PL. To the best of our knowledge, this is the first time to report on the anti-OA mechanism of PL.

## RESULTS

### Quality control of PL

**Figure 1:**
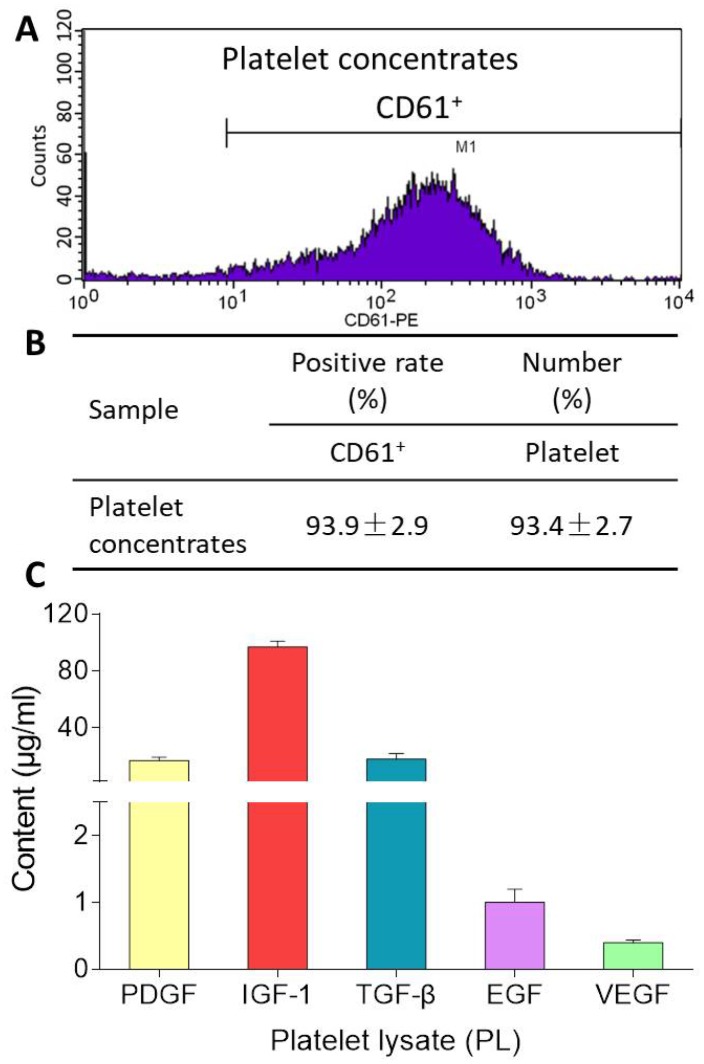
Flow cytometry pattern of platelet concentrates (**A**), CD61-positive rate and platelet number of platelet concentrates (**B**), and contents of PDGF, IGF-1, TGF-β, EGF, VEGF in PL (**C**). Values are presented as mean ± SD, *n* = 3.

CD61 is a specific cell surface marker for rat platelet. Flow cytometry analysis for CD61 surface marker expression revealed positive populations of platelet in platelet concentrates prior to PL preparation. As shown in Figure 1, the platelet concentrates contained 93.4 ± 2.7% of CD61-positive cells with CD61-positive rate of 93.9 ± 2.9%. After freeze-thaw lysis, PL was obtained and found containing 17.0 ± 2.3 μg/ml of PDGF, 96.9 ± 4.0 μg/ml of IGF-1, 18.0 ± 4.0 μg/ml of TGF-β, 1.0 ± 0.2 μg/ml of EGF, and 0.4 ± 0.04 μg/ml of VEGF.

### Anti-nociceptive effect of PL

**Figure 2:**
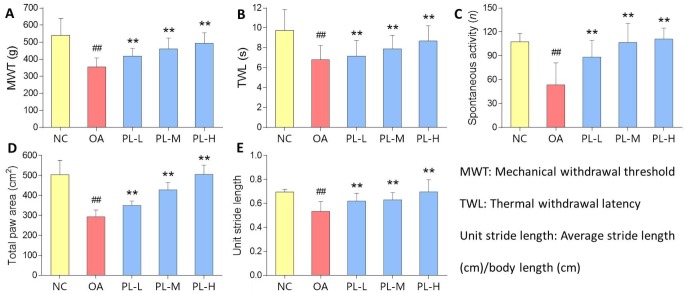
**Pain-related behavioral results of rats with PL treatment for 4 weeks.** (**A**) MWT (g); (**B**) TWL (s); (**C**) Spontaneous activity (n); (**D**) Total paw area (cm^2^); (**E**) Unit stride length. Values are shown as mean ± SD. ^##^*P* < 0.01 vs. NC group; **P < 0.01 vs. model group.

OA-induced knee pain response and anti-nociceptive effect of PL were shown in Figure 2. MWT reflected mechanical allodynia and TWL reflected thermal hyperalgesia. Spontaneous activity and gait parameters (total paw area and unit stride length) reflected pain-related behaviors. On the day 28, levels of all the parameters in the model group were significantly decreased, when compared with that of the normal group (all *P* < 0.01), indicating typical knee pain responding to arthritis modeling. When compared with the model levels, PL significantly restored the levels of above parameters toward normal levels after 28-day treatment (all *P* < 0.01).

### Therapeutic effect of PL on arthritic rats

**Figure 3:**
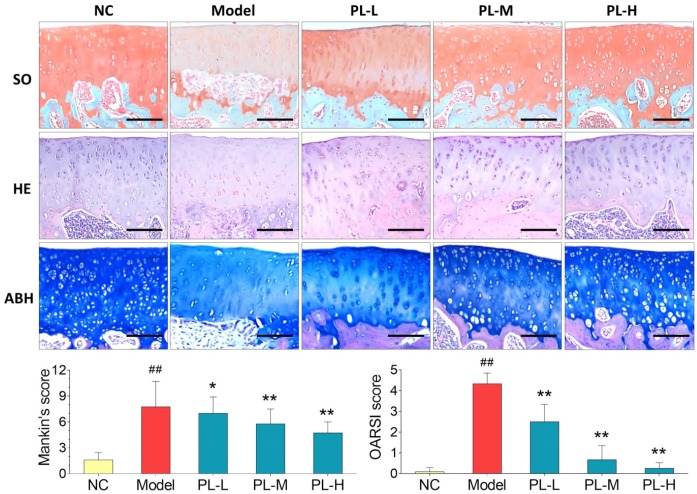
**Observation of histopathological stainings (HE, SO, and ABH) with Mankin′s scoring and OARSI scoring of rat joints.** Values are shown as mean ± SD. ^##^*P* < 0.01 vs. NC group; *P < 0.05 or **P < 0.01 vs. model group. Scale bar = 100 μm.

Results of histopathological staining were illustrated in Figure 3. Cartilage degeneration, exhibited by apoptosis of chondrocytes, loss of collagen mass, disorganization of matrix, and irregularity of cartilage surface, was seen in the arthritis model group with significantly increased Mankin′s score and OARSI score (both *P*<0.01 versus NC). In the PL-treated groups, the degeneration was gradually reversed by PL in a dose-dependent manner, with significantly decreased Mankin′s score and OARSI score (*P*<0.05 or *P*<0.01 versus model). It can be observed that the number of chondrocytes, mass of collagen in matrix, and cartilage surface were remarkably improved by increasing doses of PL.

**Figure 4:**
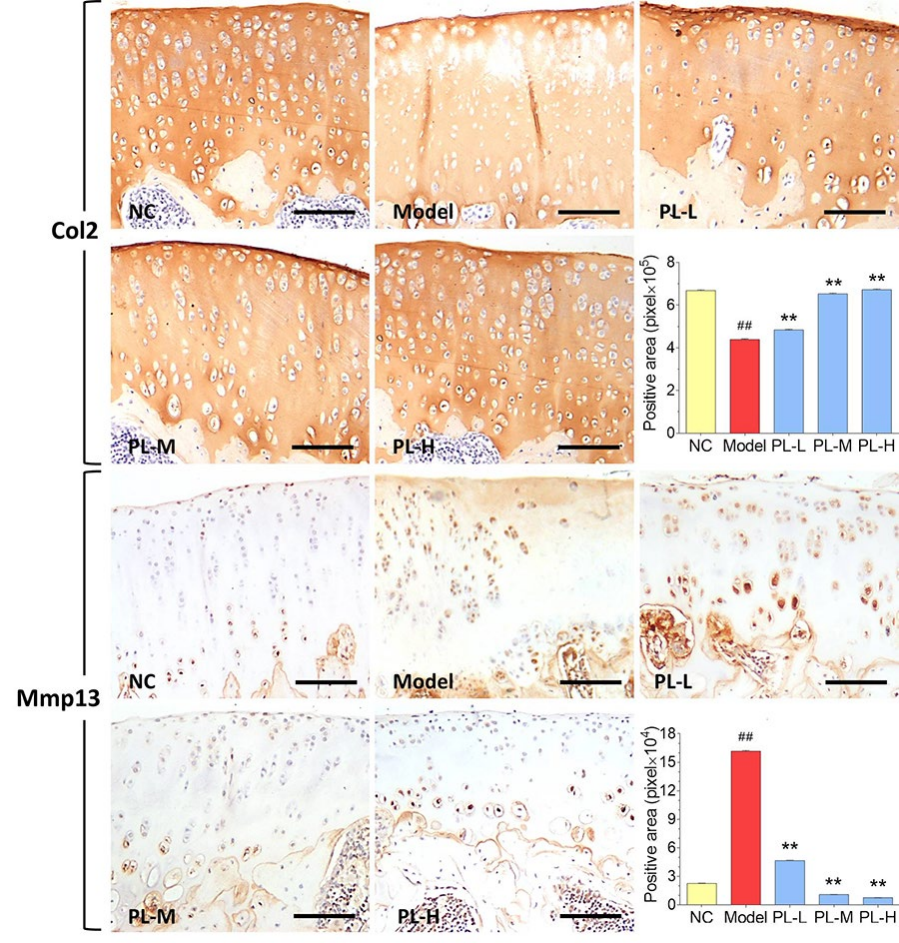
**Immunohistochemical observation and semiquantified positive area of Col2 and Mmp13 expressions in rat cartilage.** Scale bar = 100 μm.

Results of immunohistochemical analysis were shown in Figure 4. The NC group showed normal expression of Col2 in cartilage, whereas the arthritis group expressed an obvious loss of Col2 in cartilage with significant smaller positive area (*P*<0.01 versus NC). The Col2 expression was obviously upregulated and its positive area significantly increased by PL in a dose-dependent manner (all *P*<0.01 versus model), and it even reached a normal-like phenotype with PL-H treatment. Moreover, the cartilage in NC group showed almost negative Mmp13 immunoreactivity, whereas that in the arthritis model expressed stronger Mmp13 immunoreactivity with larger positive area (*P*<0.01 versus NC). The Mmp13 immunoreactivity was remarkably decreased and its positive area significantly shrank by PL treatment in a dose-dependent manner (all *P*<0.01 versus model). It can be seen that both PL-M- and PL-H-treated cartilage were almost restored to the normal phenotype.

### Cellular and molecular effects of PL on chondrocytes

**Figure 5:**
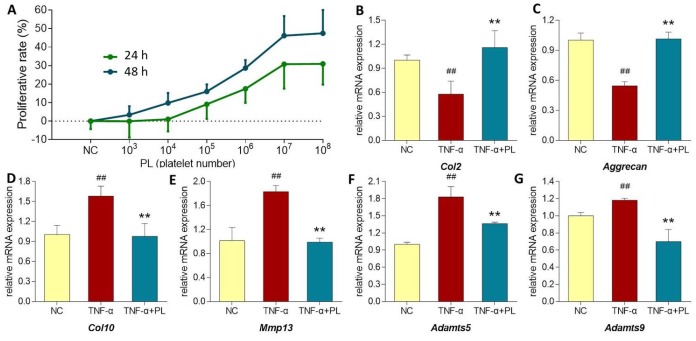
(**A**) Chondrocyte viability at 24 h and 48 h after PL treatment. (**B**–**G**) Relative mRNA expressions of target genes in chondrocytes treated with only TNF-α or TNF-α plus PL. (**B**) *Col2* expression; (**C**) *Aggrecan* expression; (**D**) *Col10* expression; (**E**) *Mmp13* expression; (**F**) *Adamts5* expression; (**G**) *Adamts9* expression. Values are shown as mean ± SD. ^##^*P* < 0.01 vs. normal cells; *P < 0.05 or **P<0.01 vs. TNF-α treated cells.

As shown in Figure 5 (upper left), PL derived from a dose range of 10^3^ to 10^8^ platelets significantly increased the chondrocyte viability with increasing proliferative rate from 0.05 ± 9.81% to 30.74 ± 13.15% at 24 h and from 3.37 ± 4.69% to 46.14 ± 10.6% at 48 h. Thus, the proliferative effect of PL on chondrocytes was dose-dependent. The regulative effects of PL on gene expressions of chondrocytes were analyzed using qPCR assay. For mimicking OA condition, chondrocytes were pretreated with TNF-α. As shown in Figure 5 (upper right and lower), TNF-α significantly downregulated the expressions of *Col2* and *aggrecan* and upregulated the expressions of *Col10*, *Mmp13*, *Adamts5*, and *Adamts9*, as compared with that of NC group (all *P* < 0.01). The altered expressions of those genes were significantly reversed by PL after 24 h treatment, as compared with that of TNF-α group (*P* < 0.05 or *P* < 0.01).

### TNF signaling pathway-related mechanism of PL

**Figure 6:**
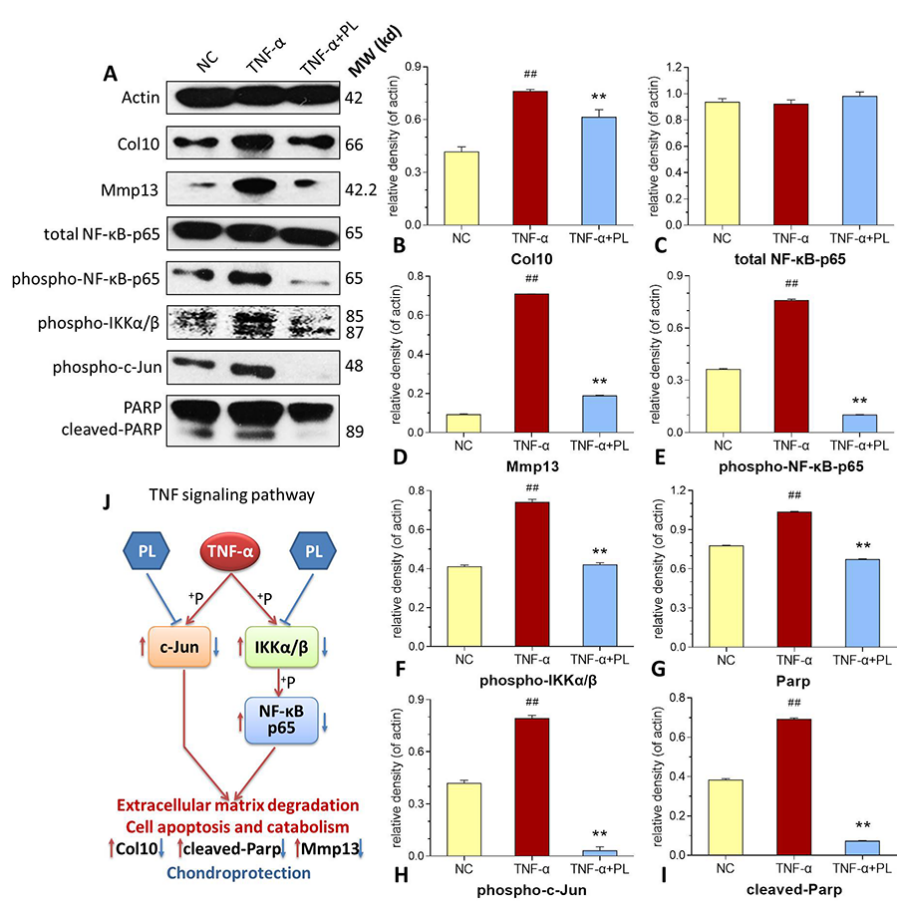
(**A**–**I**) Expression and phosphorylation of target proteins in chondrocytes. (**A**) electrophoretic profile; (**B**) Col10 expression; (**C**) total NF-κB-p65 expression; (**D**) Mmp13 expression; (**E**) phospho-NF-κB-p65 expression; (**F**) phosphor-IKKα/β expression; (**G**) PARP expression; (**H**) phosphor-c-Jun expression; (**I**) cleaved-PARP expression. (**J**) deduced process of relative TNF signaling pathway. Values are shown as mean ± SD. ^##^*P* < 0.01 vs. normal cells; *P < 0.05 or **P < 0.01 vs. TNF-α treated cells.

As shown in Figure 6, TNF-α significantly activated the phosphorylation of NF-κB p65, IKKα/β, and c-Jun (all *P* < 0.01 versus normal levels), and upregulated the protein expressions of Col10, PARP, c-PARP, and Mmp13, (all *P* < 0.01 versus normal levels). In comparison, PL significantly inhibited the phosphorylation of NF-κB p65, IKKα/β, and c-Jun (all *P* < 0.01) and downregulated the expressions of Col10, PARP, c-PARP, and Mmp13 (all *P* < 0.01) toward the normal levels.

## DISCUSSION

Aging is an important contributing factor to the development of OA, the mechanisms of which appear to be multi-factorial and may include age-related pro-inflammatory state termed “inflamm-aging” [31]. Higher levels of pro-inflammatory cytokines, such as TNF-α and IL-1β, have been shown to correlate with pain and physical function in older adults with knee OA [32]. The components of joint tissues, including the cartilage and meniscus, can be a source of pro-inflammatory mediators, in which an increased expression of pro-inflammatory cytokines was found in chondrocytes from older tissue donors with OA [33]. Breakdown of those components are handled by a set of aggrecanases (*e.g.*, a disintegrin and metalloproteinase with thrombospondin motifs, Adamts) and collagenases (matrix metalloproteases, Mmps), which are upregulated by pro-inflammatory cytokines through activation of pathways such as NF-κB in early and late OA [28, 34, 35]. These proteases are key catabolic regulators of cartilage destruction, making anti-catabolic therapy, especially that which targets pro-inflammatory cytokine-associated pathways, an attractive strategy to counteract OA. Growth factors, such as IGF-1 and PDGF, reversed the cartilage inflammation and catabolic actions by suppressing pro-inflammatory cytokine-induced transcription of the genes involved in inflammation, cartilage degradation, and chondrocyte apoptosis [16]. Therefore, growth factors may provide PL great potential in treating pro-inflammatory cytokine-associated OA.

In this study, PL was derived from concentrated platelets with high purity of >93% and contained growth factors including IGF-1, PDGF, and TGF-β (Figure 1). The data of animal experiment showed a strong anti-nociceptive effect of PL that dose-dependently reversed the MIA-induced mechanical allodynia, thermal hyperalgesia, reduced spontaneous activity, and abnormal gait patterns (Figure 2). A dose-dependent chondrocyte-protective and ECM-modifying effect was also found exerted by PL on the MIA-induced cartilage degradation (Figures 3 and 4). *In vitro*, PL upregulated the TNF-α-suppressed anabolic molecules (*Col2* and *aggrecan*) and downregulated the TNF-α-activated catabolic molecules (*Col10*, *Mmp13*, *Adamts5*, and *Adamts9*) (Figures 5 and 6). Col2 and aggrecan are the main components of cartilage, which give cartilage tensile strength and resistance to load-bearing compression [36]. Collagenase- and aggrecanase-induced degradation of Col2 and aggrecan is an early pathological marker of OA. Mmp13 acts as the major collagenase while Adamts5/9 as major aggrecanase, degrading Col2 and aggrecan in cartilage during OA progression [37, 38]. Gene deletion of the *Mmp13* or *Adamts 5* decelerated OA progression by inhibiting cartilage degeneration after surgical OA modeling [39, 40]. Col10 is a late-stage chondrocyte hypertrophy marker upregulated in OA cartilage as a result of chondrocyte hypertrophy and cartilage calcification [41]. It is associated with cartilage degradation and inflammation in patients with various degrees of OA [42]. Expressions of the above anabolic and catabolic molecules were abnormally altered by TNF-α and restored by PL, indicating that TNF signaling mediated mechanisms may have contributed to PL′s effect.

The TNF-α activated NF-κB pathway plays an important role in TNF signaling during OA cartilage destruction [43]. In this pathway, phosphorylation of IKKα/β and NF-κB p65 (p65) mediates the regulation of promoter activity of both anabolic and catabolic genes in chondrocytes. For example, phospho-p65 translocated from the cytoplasm to the nucleus, strongly activating the human *ADAMTS5* and *MMP13* activities [44, 45]. The knockdown of p65 with specific siRNA inhibited the expression of cartilage catabolic factors in chondrocytes [46]. In this study, phosphorylation of both IKKα/β and p65 were activated by TNF-α, but inhibited by PL (Figure 6). This suggests that PL rebalanced chondrocyte anabolism-catabolism through the TNF/NF-κB pathway. Moreover, our data showed that the phosphorylation of c-Jun in chondrocytes was enhanced by TNF-α, but markedly blocked by PL (Figure 6). c-Jun is a crucial downstream molecule in another TNF signaling pathway, the TNF/JNK pathway [47]. JNKs (c-Jun N-terminal kinases) are involved in chondrocyte proliferation and apoptosis as well as catabolism [48, 49]. Activated JNK phosphorylates its nuclear substrate, c-Jun, a transcription factor for the activator protein 1 (AP-1), mediating catabolic transcription and cell apoptosis/death [47, 50]. Thus, a blockage of phosphorylation on JNK or c-Jun prevents chondrocyte degradation and apoptosis/death in OA [51]. In this study, PL induced chondrocyte proliferation and blocked the phosphorylation of c-Jun and cleavage of PARP in chondrocytes (Figures 5 and 6), implying that, except for the TNF/NF-κB pathway, TNF/JNK pathway might also mediate the chondroprotective mechanism of PL. Further studies are warranted to elucidate more details of the TNF/JNK pathway mediated molecular mechanisms of PL.

Autologous platelet rich plasma (PRP) is a famous and popular non-operative treatment modality for tissue repair, especially for repairing cartilage injuries [52]. The efficacy of PRP is largely dependent on its functional components, a concentrated cocktail of growth factors stored in platelets. Findings from current studies suggest that PRP is a promising treatment for repairing cartilage defects, attenuating OA symptoms and improving joint function with an acceptable safety profile [53]. However, the efficacy of PRP for clinical applications remains unpredictable and controversial, owing to the lack of appropriate controls for validation and variable outcome data [54]. The drawbacks of PRP includes: (1) not all growth factors are released from PRP without extrinsic activation, and the heterologous activators, such as thrombin and CaCl_2_, may cause unpredictable adverse events; (2) the presence of fibrinogen and the formation of fibrin clots after thrombin stimulation may trap partial growth factors, resulting in the loss of bioactive factors; (3) highly variable methods for preparation and activation of PRP and high individual differences between different donors results in variable platelet number and growth factor content, especially when no standardized preparation and quality control was available for PRP; and (4) the storage temperature of PRP should be no less than 4 °C and the storage period is short, suggesting an immediate use after the preparation [55, 56]. PL is the next generation product of PRP, which overcomes these drawbacks by replacing PRP. It reveals the following advantages: (1) The freeze-thaw cycle is a mechanical method for activation of platelets, which avoids the use of heterologous activators and their unnecessary interference; (2) cellular debris and WBC contamination are removed during PL preparation, which remain in PRP; (3) PL contains higher contents of growth factors than that of PRP, owing to the freeze-thawing disruption of a mass of platelets; (4) it is much easier to standardize the product of PL by analyzing the growth factor content before its use, and the content is nearly independent from different donors; and (5) PL can be stored frozen for a long time, resulting in a long term use for consecutive applications [55, 56]. Without doubt, PL exerts stronger and more consistent effects compared to standard PRP′s [56]. A previous study on commercially obtained PL has reported that PL was effective in supporting monolayer expansion of FBS-free chondrocytes and proliferation of FBS-free chondrocytes in chondrogenic pellet culture [51]. Although the commercial PL led to rapid proliferation, the PL-expanded chondrocytes showed lower productions of sGAG and total collagen as well as lower gene expressions of *COL2* and *ACAN* than FBS-expanded chondrocytes, from which the authors concluded that PL might not be ideal for cell therapies [57]. However, these results were opposite to other findings that PL-expanded chondrocytes produced more sGAG than FBS-expanded chondrocytes in micromass pellets [58]. Such contradiction could be due to the difference in their preparation technics. Clearly, more work needs to be done to standardize the preparation procedure for optimal therapeutic outcome. In this study, our results showed chondroprotective effect of PL on chondrocytes, which might be steady and replicable owing to the quality control on cytokine profile we made.

Taken together, the overall results demonstrated that PL effectively ameliorated pain symptoms and prevented cartilage degradation in OA rats. The effect was achieved by the restoration of anabolism-catabolism balance through upregulation of OA-suppressed anabolic genes (*Col2* and *aggrecan*) and downregulation of OA-activated catabolic genes (*Col10*, *Mmp13*, *Adamts5*, and *Adamts9*) in chondrocytes. Its molecular mechanism was associated with inhibition of NF-κB signaling and blockage of c-Jun signaling in the TNF signaling pathway. This study provides certain knowledge of anti-OA effect and mechanism of PL, placing it as promising and alternative therapeutic option for OA therapy in the future.

## MATERIALS AND METHODS

### Chemicals and reagents

IMDM (Iscove’s modified Dulbecco's medium) and trypsin (0.25%) were purchased from Thermo Fisher Scientific (MA, USA). FBS (fetal bovine serum) was purchased from CellMax (Beijing, China). MTT (3-(4,5-Dimethylthiazol-2-yl)-2,5-diphenyltetrazolium bromide) and DMSO (dimethyl sulfoxide) were purchased from Sigma-Aldrich (Taufkirchen, Germany). MIA was purchased from Sigma-Aldrich. The cell cycle kit was obtained from BD Biosciences (San Jose, CA, USA). Real time PCR (polymerase chain reaction) kit and TRIzol reagent were purchased from TaKaRa Biotechnology Co. Ltd. (Dalian, China). All primary antibodies were purchased from Cell Signaling Technology Inc. (Danvers, MA, USA). All ELISA kits were purchased from Multi Sciences (Lianke) Biotech Co., Ltd (Hangzhou, China).

### Platelet concentrate preparation and PL extraction

Platelet lysate (PL) was obtained by lysing platelet concentrates of rat plasma according to the acknowledged method (2-step centrifugation procedure) with modifications [59]. Briefly, anti-coagulant whole blood (3% v/v sodium citrate) was treated by sequential rounds of centrifugation at 4 °C (10 min at 210 × *g* and 5 min for 210 × *g*), with the non-erythrocyte volume collected subsequently to each round. The collected buffy coat was washed three times with PBS and concentrated through supernatant removal to obtain platelet concentrates. The final platelet number was measured by Mindray BC-3000plus blood cell analyzer (Shenzhen, China) and standardized to 1 × 10^8^ platelets/ml. The platelet concentrate was lysed by repeating a freeze-thaw (-80°C to 37°C) three times, followed by centrifugation at 2,000 × *g* for 10 min to remove remaining platelet fragments. The obtained supernatant containing bioactive growth factors (PL) was divided into aliquots and stored at -80°C before use.

### Immunophenotypic analysis and enzyme-linked immunosorbent assay (ELISA)

The expression of platelet surface marker in platelet concentrates was assessed by flow cytometry analysis. Before PL extraction, the platelet concentrates were suspended and incubated with the fluorochrome-conjugated antibody (anti-CD61-PE) against rat antigen for 1 h in the dark at RT. After incubation, PL was washed with PBS three times and re-suspended in PBS for flow cytometry analysis (BD FACSCalibur, BD Biosciences, CA, USA) in triplicates. Fluorescent signal intensity was recorded and analyzed by CellQuest software. After PL extraction, the concentrations of epidermal growth factor (EGF), insulin-like growth factor (IGF-1), platelet-derived growth factor (PDGF), transformating growth factor β (TGF-β), and vascular endothelial growth factor (VEGF) of PL were measured in triplicate using commercially available ELISA kits (Lianke Biotech Co., Hangzhou, China), in accordance with each manufacturer’s instructions. The absorbance was measured using a microplate reader (Bio-Rad Laboratories, Inc., Hercules, CA, USA).

### Animal and animal experiments

Male Sprague Dawley (SD) rats (Grade SPF II) with body weight of 200 ± 20 g were provided by Shanghai Super B&K Laboratory Animal Co. Ltd. (Certificate number: SCXK (Shanghai) 2013-0016). All rats were housed in cages under pathogen-free condition with 12 h light/dark cycle and provided with food and water ad libitum. The animal experiments were in accordance with the China legislation on the use and care of laboratory animals and approved by the Medical Norms and Ethics Committee of Zhejiang Chinese Medical University.

Fifty rats were equally and randomly divided into five groups: NC group as normal control group, model group as arthritis model group, PL-L as low dose of PL (10^5^ platelet-derived PL) treated model group, PL-M as middle dose of PL (10^6^ platelet-derived PL) treated model group, and PL-H as high dose of PL (10^7^ platelet-derived PL) treated model group. The NC group was treated with 50 μl of saline by intra-articular injection. The model group and all PL groups were treated with 50 μl of MIA (30 mg/ml) by intra-articular injection for modeling of arthritis. After seven days, PL-L, PL-M, and PL-H groups were treated with 50 μl of 10^5^ platelet-derived PL, 10^6^ platelet-derived PL, and 10^7^ platelet-derived PL, respectively, by weekly intra-articular injection. Meanwhile, NC group was treated with 50 μl of saline in a same route. After the treatments for four weeks, mechanical withdrawal threshold (MWT), thermal withdrawal latency (TWL), spontaneous activity, and treadmill gait analyses were measured. At the end of the experiment, all rats were euthanized by over anesthesia. Immediately, articular samples were taken from their knee joints for histopathological observation and immunohistochemical analysis.

### Pain behavior and gait patterns analyses

The MWT, TWL, and spontaneous activity were measured as previously described [60]. Briefly, each rat was placed in a wire-mesh based cage for 30 min-acclimatization, followed by needling at its plantar surface of hind paws for three times. The needle pressure (*g*) which caused paw withdrawal was recorded as MWT. A focused beam of radiant heat was irradiated to the rat plantar surface of hind paws for three times, and the time length (s) before the heat-caused paw withdrawal was recorded as TWL. Each rat was placed in a dark box equipped with a monitor system for recording its activity, and the activity times within 10 min was recorded as the spontaneous activity parameter. The gait patterns of each rat, including paw area (cm^2^), stride length (cm), and body length (cm), were measured using the DigiGait System and the data captured by a video recorder (DigiGait Imaging System, Mouse Specifics, Boston, MA, USA). Total paw area (cm^2^), average stride length (cm) and Unite stride length were calculated.

### Histopathological observation and immunohistochemical analysis

Each articular sample was fixed with formalin (10%) for 24 h and decalcified with EDTA (10%) in PBS for eight weeks. Then each sample was embedded in paraffin and sectioned into 2-3 μm, followed by staining with HE (hematoxylin and eosin), SO (safranin-O), or ABH (alcian blue/hematoxylin). The stained sections were observed under microscopy and statistically graded on a scale of 0−13 by double-blind observation, according to the Mankin's scoring and OARSI scoring systems [61, 62]. Unstained replicates of the sections were incubated overnight at 4 °C with 100 μl PBS-diluted (1:100) primary antibodies against rat Col2 (rabbit anti-Col2 monoclonal antibody) and rat matrix metalloproteinase 13 (mouse anti-Mmp13 monoclonal antibody). After PBS wash, the sections were incubated with Horseradish peroxidase (HRP) conjuncted secondary antibodies (PV-9001 for Col2 and PV-9002 for Mmp13) (ZSGQ-BIO, Beijing, China) for 20 min at room temperature, followed by colorimetric detection using 3,3′-diaminobenzidine (DAB) substrate chromogen for 8 min. The immunoreactivity of Col2 and Mmp13 were semiquantified by using Image-Pro Plus (IPP) 6.0 software (Media Cybernetics, Bethesda, MD, USA) under a light microscope (NIKON 80i, Tokyo, Japan).

### Primary chondrocytes

Primary chondrocytes were obtained from allogeneic male SD rat donors as previously described [63]. Briefly, articular cartilage tissues from four rat donors were harvested and sliced into small pieces. The pieces were digested with 0.25% trypsin for 40 min at 37°C and then with 0.1% Col2 for 4 h at 37°C. The isolated cells were filtered through a cell strainer (70 μm) and collected. The cells were identified as chondrocytes by morphology, toluidine blue staining and Col2 immunocytochemical staining. IMDM medium containing 10% FBS was used to culture the chondrocytes.

### Cell viability assay

The cell viability of chondrocytes was evaluated by MTT assay, as previously described [60]. Briefly, the chondrocytes were seeded on 96-well plates for 24 h and treated with PLs derived from 10^3^, 10^4^, 10^5^, 10^6^, 10^7^, and 10^8^ platelets for another 24 h and 48 h. MTT solution was added to each well and incubated for 4 h. Subsequently, DMSO was added into each well to dissolve the formazan crystals. The optical density (OD) value was measured at 490 nm by a Bio-Rad microplate reader (Hercules, CA, USA). Proliferative rate (%) = (PL-treated OD/untreated OD) × 100.

### Real time PCR (qPCR) assay

The primary chondrocytes were divided into three groups as follows: NC group was the normal control cultured for 30 h with no treatment; TNF-α group was the model group pre-treated with TNF-α (10 ng/ml) for 6 h and then cultured for 24 h with no further treatment; and TNF-α+PL group was the treatment group pre-treated with TNF-α (10 ng/ml) for 6 h and then treated with PL (derived from 10^7^ platelets) for 24 h. Total RNA was extracted with TRIzol reagent and quality controlled by NanoDrop2000 spectrophotometer (Thermo Scientific, USA) and agarose gel electrophoresis. The quality of all RNA samples was verified with good purity and integrity before use. Then, the reverse transcription was conducted to produce cDNA. As previously applied, the final qPCR reaction system was 20 μl, comprising 10 μl SYBR^®^ Premix Ex Taq II (Tli RnaseH Plus), 0.4 μl PCR Forward Primer, 0.4 μl PCR Reverse Primer, 1 μl template cDNA and 8.2 μl ddH_2_O, and the qPCR reaction conditions were as follows: 95°C for 5 min for initial denaturation, followed by 40 cycles of denaturation at 95°C for 10 sec, annealing and extension at 60°C for 30 sec [60]. The qPCR assay was performed on an ABI QuantStudio^TM^ 7 Flex Real-Time PCR System (Applied Biosystems; Thermo Scientific, USA). β-Actin was used as the reference gene and 2^-ΔΔ CT^ method was applied to measure the relative mRNA expression (Table 1).

**Table 1:** Primer sequences of target genes

**Gene**	**Forward primer**	**Reverse primer**
β-actin	5′-CCCGCGAGTACAACCTTCT-3′	5′-CGTCATCCATGGCGAACT-3′
*Col2*	5′-CTCAAGTCGCTGAACAACCA-3′	5′-GTCTCCGCTCTTCCACTCTG-3′
*Col10*	5′-GATCATGGAGCTCACGGAAAA-3′	5′-CCGTTCGATTCCGCATTG-3′
*Mmp13*	5′-CTATGGTCCAGGAGATGAAGAC-3′	5′-GTGCAGACGCCAGAAGAATCT-3′
*Adamts5*	5′-TGGAGTGTGTGGAGGGGATA-3′	5′-CGGACTTTTATGTGGGTTGC-3′
*Adamts9*	5′-TACAGGCAAAGGCTGGTCTC-3′	5′-CTCAGGTAGCAGGGATGGAC-3′
*Aggrecan*	5′-GCAGACATTGATGAGTGCCTC-3′	5′-CTCACACAGGTCCCCTCTGT-3′

### Western blot (WB) analysis

The chondrocytes were grouped and treated in accordance with qPCR assay procedure. As previously described, the total proteins were extracted by using lysis buffer (50 mM Tris-HCl, pH 7.4, 150 mM NaCl, 1 mM EDTA, 1% Triton and 0.1% SDS) with proteinase inhibitor cocktail (Bimake, Houston, TX, USA) for 30 min on ice [64]. Proteins were separated by SDS-PAGE (6-12%) and transferred onto a nitrocellulose membrane (Sartorius Stedim, Göttingen, Germany). The membrane was blocked with 5% non-fat milk for 2 h, followed by overnight incubation at 4°C with primary antibodies including actin, Col10, Mmp13, total NF-κB p65, phosphor-NF-κB p65, phosphor-IKKα/β, phosphor-c-Jun, poly (ADP-ribose) polymerase (PARP), and cleaved-PARP. Following incubation with peroxidase-conjugated goat anti-rabbit/mouse IgG at room temperature for 2 h, proteins were visualized using Western Lightning^®^ Plus ECL (Perkin Elmer, Inc., Waltham, MA. USA), detected using X-ray film (Kodak, Tokyo, Japan) and scanned [64].

### Statistical analysis

Data were expressed as the mean ± standard deviation (SD). Data from different groups were compared using one-way ANOVA followed by Fisher’s least significant difference (LSD) comparison. A *p*-value < 0.05 was considered to indicate a significant difference and *p*-value < 0.01 considered to indicate a very significant difference. The Mankin′s score and OARSI score were assessed by three independent observers in a blind manner. All analyses were performed using an updated version of DPS software [65].
